# Research on the Environmental Philosophy of China’s Environmental Crime Legislation from the Perspective of Ecological Civilization Construction

**DOI:** 10.3390/ijerph20021517

**Published:** 2023-01-13

**Authors:** Ran An, Peng Liu

**Affiliations:** 1 Law School, Qufu Normal University, Rizhao 276826, China; 2 Intellectual Property Law and Policy Institute, East China University of Political Science and Law, Shanghai 200042, China

**Keywords:** environmental crime, environmental philosophy, anthropocentrism, ecocentrism, ecological civilization

## Abstract

Modern environmental philosophy is a new type of philosophy for humans re-examining the relationship between man and nature and provides the value guidance for modern environmental law. China’s environmental crime legislation has gone through the exploration period, establishment period, and optimization period. The environmental philosophy behind this is worth discussing and determines the direction China will take environmental crime in the future and whether China’s environmental strategy can really be implemented. At present, the disputes about the environmental philosophy of environmental crime in China are mainly reflected in the contention between anthropocentrism, ecocentrism, and eco-anthropocentrism. There are radical risks of pure human centrism or pure ecological centrism, and these two theories struggle to serve as a value basis for environmental crime legislation. Although eco-anthropocentrism seems to be comprehensive, it is actually ambiguous, and it is still difficult to deal with the conflict between people and nature. In recent years, China has continuously emphasized the construction of ecological civilization construction and written this into the constitution. Therefore, in the environmental philosophy issues of environmental crimes in China, we should consider absorbing the advantages of anthropocentrism, ecocentrism, and eco-anthropocentrism, while taking the original Chinese ecological civilization philosophy as the value foundation.

## 1. Introduction

It is generally believed that philosophy is a summary of the basic issues of the world and the theoretical system about the worldview. Environmental philosophy is a philosophical field that explores the relationship between humans and the environment, which deeply affects a country’s attitude towards environmental protection and its environmental governance policies and legal management [1,2]. In ancient times, human beings also had an environmental philosophy, but in the context of the current environmental policy formulation and academic research around the world, environmental philosophy mainly refers to the environmental philosophy spawned by environmental crises that emerged after the 1940–1950s [3,4]. The main genres include anthropocentrism and non-anthropocentrism, and the latter includes animal rights theory, biological center theory, ecological center theory, etc. [5]. Modern environmental philosophy has greatly deepened the understanding of human relations with nature and interpersonal relationships and has made important contributions to the social development of post-industrial civilization [6].

As is known to all, China has long been a world factory, and it has inevitably become the world’s largest greenhouse gas emission country. China’s environmental protection is not only related to the healthy life of the Chinese people, but also a key factor concerning the success of the world’s environmental protection, which has attracted the attention of the world. Since the 1990s, modern environmental philosophy-related monographs and papers have been introduced into China in large numbers, which have played a major enlightenment and supporting role in the development of China’s research on environmental philosophy and environmental politics [7,8,9,10,11,12]. Several views and initiatives regarding environmental philosophy have been heatedly discussed in environmental law research in China. However, since China promulgated the “Environmental Protection Law” in 1989, until the first 10 years of the 21st century, in more than a 30-year span, the development of China’s environmental law is more reflected in the promulgation of more environmental laws. Because the enforcement of these environmental protection laws is not ideal, the environmental crisis in China has continued to deteriorate.

This situation has been significantly improved in the past ten years. The most important reason is that China has substantially modified environmental criminal law and has greatly strengthened the application of these crimes. Criminal law is the most powerful guarantee of a nation’s social governance; thus, environmental crime legislation is a nation’s ballast in the field of environmental governance [13]. Unlike the United States and the United Kingdom, who set environmental criminal clauses in environmental law, China, Germany, and a lot of civil law countries set environmental criminal clauses in the criminal code. Due to the deprivation of the defendant’s freedom or even life, the changes of the Criminal Law Code are often very serious. In recent years, China’s environmental crime legislation has been amended several times, which has played an irreplaceable role in the improvement in China’s environmental governance and environmental consciousness. This has become one of the most popular areas in China’s criminal law research at present [14,15,16]. This article will show that the huge changes in China’s environmental criminal law are a concentrated manifestation of China’s dedication to environmental protection. Therefore, the vicissitude and development of environmental philosophy behind China’s environmental criminal law are worthy of research.

In recent years, the international community’s attention on Chinese legal issues has continued to increase, but there is relatively little international literature on China’s environmental criminal law, and the themes of existing literature are scattered. Few articles paid attention to environmental philosophy issues of environmental criminal law in China. References [17,18,19,20,21,22,23] Unlike many environmental protection pioneer countries, which have long been using criminal law to combat environmental crime, China’s environmental crime legislation was not promulgated until China’s new criminal code came out in 1997 [24]. The environmental criminal law was also amended and improved in subsequent amendments. The most important provision is Article 338, the crime of major accidental environmental pollution. However, as this legislation was enacted at the end of the 20th century, when China was still in the stage of rapid economic development and people’s environmental awareness was weak, the subjective fault of this charge is negligence; in other words, intentional damage to the environment does not constitute this crime. This makes the application of this provision extremely difficult, and this provision fails to play a significant role in environmental protection. It is gratifying that, in the Amendment (VIII) to the Criminal Law, the name of the crime was changed to “environment pollution crime”, which incriminates intentional pollution of the environment, and on June 18, 2013, China’s Supreme People’s Court and the China’s Supreme Procuratorate jointly issued the Explanation in Handling Criminal Cases of Environmental Pollution, clearly setting quantitative criteria for the environmental pollution crime. As a result, the number of environmental pollution crimes has seen a surge in recent years.

When the provision for environmental crime was first established in the Criminal Law in 1997, the provision could not combat intentional acts of environmental pollution and, therefore, could not serve the purpose of environmental protection. It was criticized by the academic community as a type of legislation guided by “anthropocentric values” and was deemed to fail to recognize and protect the value of nature [25,26]. Since then, the Chinese criminal law academia has been debating whether environmental legislation should protect the interests of people or protect the interests of nature itself. The two sides have often quoted the views of environmental philosophy theories, showing the trend of turning legal issues into environmental philosophy issues [27]. In 2011, China changed the crime of “major environmental pollution accident” to “environment pollution crime “. Some scholars believe that this change reflects that environmental crime legislation in China is shifting from anthropocentrism to an environment-based philosophy [28]. In fact, before the implementation of Amendment VIII, the academic community had been calling for the abandonment of anthropocentrism in environmental crime legislation [29]. However, there are also a number of scholars who refute this view, and even openly express support for anthropocentrism. Both sides stuck to their argument and would not give in [30].

In general, philosophy refers to the human views, ideas, and beliefs regarding certain things. Legislation philosophy can be summarized as humans’ understanding, ideology, consciousness, theory, rationality, and ideals for legislation, but also includes the characterization of these thinking products, such as legislative purposes, objectives, tenets, principles, norms, and pursuits [31]. Legislation, as a human practice, is undoubtedly performed under the governance of legislation philosophy, which can be deemed the guiding ideology and soul of legislation [32]. The legislation philosophy of environmental crime can guide theoretical research and legal practice and is, therefore, of paramount significance. We believe that, as China’s efforts in environmental crime prevention and control continue to enhance, there will be more “pure environment-damaging activities” in the eyes of anthropocentrism critics that are considered environmental crime, and the debate on the different interpretations of environmental legislation philosophy will continue. 

Meanwhile, the status of ecological civilization construction in China’s national development strategy has become increasingly important since 2007 and has now become one of the five major features of socialism with Chinese characteristics. Moreover, the national development strategy of ecological civilization construction was written into the Chinese Constitution in 2018 [33]. Therefore, in the context of China’s current vigorous promotion of the construction of an ecological civilization, its similarities and differences with modern environmental philosophy are worth discussing. The kind of value guidance for China’s environmental crime and justice that will occur in the future is the research focus of this article. The author will explore this issue in the following four sections, using the comparative research method. This article will first sort out the different characteristics of China’s environmental crime legislation in different periods, as well as the main data of environmental crime legislation in the judicial practice in the last 20 years. Then, this article will sort out the environmental philosophy disputes reflected in the environmental crime legislation. Subsequently, this article will study environmental crime legislation in the United States, Germany, France, and Japan for reference. Finally, this article will put forward suggestions regarding the values that China’s environmental crime legislation and justice should adopt.

## 2. Legislative Development and Judicial Status Quo of Environmental Crimes in China

Although there are also elements related to the natural environment in the crimes in other chapters, the legislation of China’s environmental crimes mainly refers to the 16 crimes stipulated in Chapter VI Section VI of the Chinese Criminal Law. The 16 crimes can be roughly divided into environmental pollution (such as the Crime of Environmental Pollution), resource destruction (such as the Crime of Illegal Mining), and harm to animals and plants (such as the Crime of Illegal Hunting). The exploration of China’s environmental crime legislation began in 1979 and was formally established when the criminal law was fundamentally amended in 1997. After 2011, the revision of China’s environmental crime legislation has entered a more frequent rhythm.

### 2.1. Legislative Development of Environmental Crimes in China

#### 2.1.1. Comprehensive Exploration Period

In the long period after the founding of The People’s Republic of China, the issue of environmental protection did not attract the attention of the government and the public, and the number of regulations on environmental crimes were few and could be ignored. After entering the 1970s, China began to seriously think about environmental protection issues. In 1972, China actively participated in the Human Environment Conference held in Stockholm, which was the first important international conference in which China participated after regaining its legitimate seat in the United Nations. Since then, the issue of environmental protection has attracted real attention from the Chinese government [34]. Subsequently, China’s 1978 Constitution expressed the statements that the state protects the environment and natural resources and prevents pollution and other public hazards. In this context, China’s environmental crime legislation entered a period of comprehensive exploration. At this stage, China’s criminal code included the crime of illegal logging, illegal hunting, and other crimes, and the crime of illegally hunting and killing precious and endangered wild animals was stipulated in the form of a single criminal law. In addition, criminal provisions were subordinately stipulated in environmental laws such as the Water Pollution Prevention and Control Law, Marine Environmental Protection Law, the Mineral Resources Law, and the Air Pollution Prevention and Control Law [35]. China’s environmental crime legislation at this stage reflects China’s preliminary emphasis on environmental protection issues—the crimes cover many fields of environmental protection with diversified legislative forms—which laid the foundation for the formal establishment of environmental crime legislation. China’s environmental crime legislation, which was in the exploratory period, also reflected the characteristics of fragmentation and light punishment. Environmental crime was not a main crime in China’s criminal law in this historical period nor did it attract enough attention from public society.

#### 2.1.2. Formal Establishment Period

In 1997, China’s Criminal Code underwent a comprehensive overhaul, and the environmental crime legislation was formally established in this period. In the sixth chapter of China’s new criminal code, Crimes of Obstructing Social Management Order, environmental crimes were given an exclusive section, namely, the Crimes of Destroying the Protection of Environmental Resources in the sixth section. At this stage, China’s environmental crime legislation had three advanced aspects. First, professional environmental pollution crimes entered the Criminal Code. Before the promulgation of China’s new criminal code, China’s environmental crime legislation mainly focused on mineral destruction, forest destruction, illegal hunting of wild animals, etc., while professional environmental pollution crimes were few. The reason for this may be that the environmental pollution crisis in China was not very serious at that time and may also be that the Chinese government and the public did not have a deep understanding of the harm caused by environmental pollution. After China’s reform and opening up in 1978, China’s economy developed rapidly, and by the time a new criminal code was compiled, China’s environmental crisis was already very dangerous [36]. Therefore, the Crime of Major Environmental Pollution Accident was added to the new criminal law to curb the environmental crisis. Additionally, the scope of criminal subjects was expanded, and unit crimes were added to the new criminal code. Article 346 of the Chinese Criminal Code stipulates that if a unit commits the crimes specified in Articles 338–345 of this section, the unit shall be sentenced to a fine, and the directly responsible person in charge and other directly responsible personnel shall be punished according to the respective provisions, which fills previous legal loopholes. The reason for this is that environmental crimes are mainly for the purpose of profit-making, and there are many entities in the unit that are involved, so if only the person in charge of the unit is punished, it is often impossible to obtain sufficient funds for environmental restoration. By adding punishment provisions for the main body of the unit, the polluted natural environment can be more effectively recovered. In another vein, the statutory penalty for environmental crimes has been increased. For example, the new Criminal Code raised the statutory maximum penalty for the crime of deforestation to seven years in prison, and the statutory maximum penalty for the crime of illegal logging was raised to 15 years in prison, which significantly increased the deterrent effect of environmental criminal law. China has enacted special legislation regarding environmental crimes in the new Criminal Code, which ended the fragmentation of China’s environmental crime legislation and further reflects China’s emphasis on environmental protection.

#### 2.1.3. Active Optimization Period

The formal establishment of environmental crimes in China’s new criminal law provides a solid guarantee for China’s environmental protection cause, but at the end of the 20th century and the beginning of the 21st century, economic construction has still been the core goal of China’s national development strategy. Therefore, there are not as many environmental crime filings as expected, and they have not fulfilled the due function of environmental crime legislation [37,38]. In contrast, there have been relatively more judicial judgments for crimes, such as illegal logging, illegal hunting, and illegal mining, but very few decisions for major environmental pollution accidents, which is one of the critical reasons for the intensifying environmental pollution crisis in China [39]. In this context, China formally proposed the national strategy of building an ecological civilization in 2007, which would increase the protection of the ecological environment. Meanwhile, with the continuous improvement in the standard of education of the Chinese people and the continuous increase in the number of the middle class, Chinese citizens’ awareness of environmental protection has significantly increased, and the requirements for environmental protection have become increasingly strong. Therefore, the Criminal Law Amendment (VIII), which was introduced in 2011, adjusted the Crime of Major Environmental Pollution Accident to the Crime of Environmental Pollution, which significantly lowered the threshold for criminalization for environmental pollution behavior. This drastically strengthened the practical effects of the charge. During the period from 2011 to 2021, the number of decisions for environmental pollution crimes exceeded 12,000, which significantly enhanced the hardness and strength of China’s environmental protection cause. During this period, China wrote the construction of ecological civilization into the Chinese constitution, and Chinese leaders further pointed out that China should implement the strictest environmental protection system [40]. Therefore, the Criminal Law Amendment (eleven) was promulgated in 2021, which further strengthened China’s environmental crime legislation. First, the statutory penalty for environmental pollution crimes was increased from the original maximum of seven years to more than seven years, which makes environmental pollution behavior punishable with a maximum penalty of 15 years in prison, demonstrating China’s determination to strengthen environmental protection. Second, the Criminal Law Amendment in 2021 included the consumption of all terrestrial wild animals within the scope of punishment in Criminal Law, significantly expanding the circle of protection for wild animals. Third, the latest environmental crime legislation also included illegal commercial development and the construction of buildings in national nature reserves within the scope of punishment, expanding the scope of protection of the natural environment.

Through the exploration and development that has occurred over these 40 years, China’s environmental crime legislation has preliminarily formed a system. With more and more judiciary attention being paid to the application of environmental crime clauses, the number of environmental crime proceedings continues to grow. This legislation plays an irreplaceable and critical role in the improvement in China’s natural environment.

### 2.2. Judicial Status of Environmental Crimes in China

**Figure 1:**
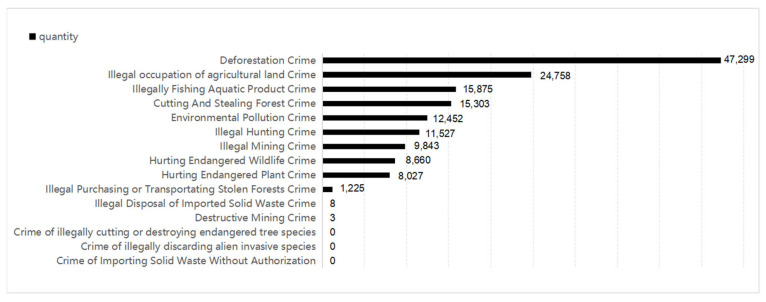
Number of judgements on various environmental crimes in China.

By searching the China Judgment Online hosted by the Supreme People’s Court of China, the author will show the number of judgments on 15 charges of environmental crimes in China, as shown in Figure 1 below. (It should be noted that China Judgment Online is the most authoritative database of judicial judgments in China, which mainly collects judicial instruments from courts at all levels in China. Since the 21st century, this database has collected 136 million copies [41].) The environmental crime judgment data shown in Figure 1 show the approximate number of judgments from the last 20 years in China, without including data from the late 1990s.

It can be seen from Figure 1 that, in the last 20 years, 152,989 environmental crime cases were decided in China, which is a considerable number. The quantity gap between different charges is notable. For instance, there were six charges in more than 10,000 cases, while six crimes had fewer than eight cases, and, among these, there were three crimes with zero judgment. Figure 1 shows that China’s protection of natural resources is very worthy of recognition: there are more than 60,000 cases related to forest resources, more than 36,000 cases related to wildlife protection, and more than 20,000 cases related to land. In contrast, there are over 12,000 environmental pollution crimes, which seems to indicate that China does not pay enough attention to the crime of environmental pollution. When the data for this crime are listed separately, however, China’s punishment for environmental pollution crimes in recent years is shown to greatly exceed that of the first 10 years of the 21st century.

**Figure 2:**
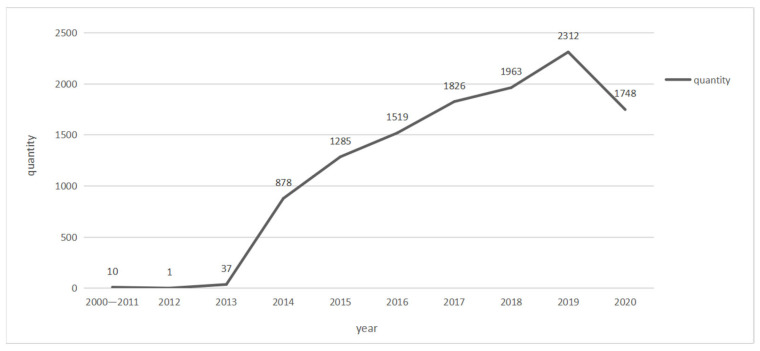
Growth trend of cases of Article 338 of China Criminal Code (2000–2022).

Figure 2 shows the number of cases under Article 338 of the Criminal Law of China in the last 20 years. Before 2011, the crime of Article 338 is named Crime of Major Accidental Environmental Pollution, which was almost not applicable in the first 10 years of the 21st century, and it can even be said that it became a zombie clause. After the crime was adjusted to the Crime of Environmental Pollution in 2011, especially after the Supreme People’s Court and the Supreme People’s Procuratorate jointly issued a relevant judicial interpretation in 2013, the Crime of Environmental Pollution was applied on a large scale. Since environmental pollution crime cases are closer to people’s lives, after a large number of environmental pollution crime cases were sentenced by judicial courts, they have greatly deterred various subjects of environmental pollution. This has enhanced the sense of the existence of environmental crimes in China, playing a significant role in improving the environmental awareness of the Chinese public.

**Figure 3:**
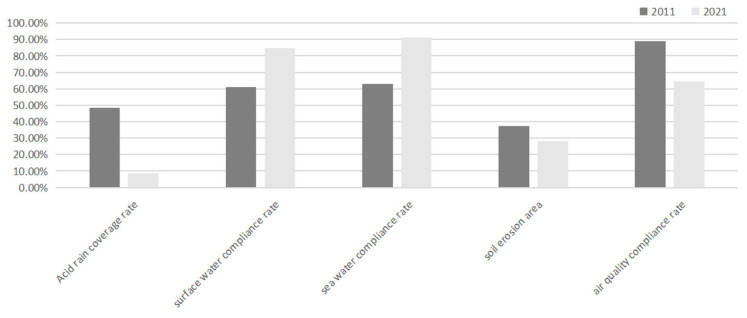
Comparison of the main indicators of environmental quality in China.

Thanks to the protection and guarantee of China’s environmental crime legislation and judiciary, as well as the implementation of other environmental governance measures, China’s environmental crisis, which began at the end of the 20th century, has finally stopped deteriorating, and China’s natural environment has significantly improved. Figure 3 shows a comparison of the main indicators of China’s environmental quality in 2011 and 2021. The data come from the China Environmental Bulletin, issued annually by the Chinese Ministry of Ecology and Environment.

As can be seen from Figure 3, China’s acid rain coverage (China’s land area covered by acid rain) has decreased by 40%, soil erosion area rate (soil erosion area accounting for China’s land area) has decreased by 10%, surface water compliance rate has increased by 23.9%, and sea water compliance rate has increased by 28.5%. The only regret is that China’s atmospheric compliance rate has decreased by 24.7%, which also echoes the severe reality that China is currently the world’s largest emitter [42]. China’s achievements in environmental governance should be appreciated. However, China still shoulders heavy environmental burdens and is still facing the world’s greatest pressure to reduce emissions. In this context, it is worth discussing what kind of environmental philosophy should guide the legislation and judiciary regarding environmental crimes in China in the future.

## 3. Evolution of Environmental Philosophy in China’s Environmental Crime Legislation

Throughout the whole process of China’s environmental crime legislation, from the exploration period to the active period, the environmental philosophy of its legislation has gradually evolved. During the exploration period of China’s environmental crime legislation, the consideration of environmental protection issues by the legislators was very elementary; it can be said that the exploration of environmental crime legislation started in a state of enlightenment. After China’s environmental crime laws were established by the new criminal law, the debate on environmental philosophical issues of China’s environmental crime legislation gradually became a fierce academic debate.

The reason for this is that, before 2011, China’s environmental crime legislation was considered to reflect anthropocentrism in general, and the punishment for the environmental pollution crimes of potential environmental damage gave this legislation an ecocentric implication. At present, the discussion of environmental philosophy in China’s environmental crime legislation is mainly reflected in the contention between anthropocentrism, ecocentrism, and eco-anthropocentrism [43].

### 3.1. The Theory of Anthropocentrism

Anthropocentrism is a summary of the modern environmental philosophical theory on the traditional human-focused outlook, values, and philosophy regarding the relationship between man and nature. In China’s theoretical field, however, there are still many scholars who insist that China’s environmental crime legislation should adhere to the anthropocentric environmental philosophy [44].

The anthropocentric environmental philosophy supported by these scholars does not mean that human beings are the absolute center of the world, but that the science and technology developed by humans give humans the ability to use natural resources to have a better life, which means that the situation where human beings are powerless to nature has gone forever. No longer forced to yield to the energy of nature, human beings have full capacity to change and utilize nature and become governors of nature [45]. Under the guidance of this environmental philosophy, many scholars believe that the reason the natural environment should become the object of criminal law protection is that the environment can ultimately be associated with individual legal interests; thus, criminal environmental pollution must threaten human life, health, or property. In other words, the ultimate purpose of the crime of environmental pollution is not to protect the environment, but human life, health, and property. This suggests that the natural environment itself is not an independent legal interest, but only a condition for human development [30].

The theoretical advantage of anthropocentric legislative philosophy lies in its clear inheritance of the spirit of modern liberal criminal law, which advocates restricting the activation of the right to punish. The theory holds that only when environmental pollution has actual impact on personal life, health, and legal interests regarding property, or posts a serious threat at an empirical level, can criminal law can be applied. This theoretical proposition undoubtedly better protects the freedom and rights of citizens and can more easily gain public understanding [46]. However, the shortcoming of this theory is that theoretical support for the current legislation and justice regarding environmental crime is very limited. The judicial practice regarding environmental pollution crimes in recent years shows that air pollution and water pollution cases account for a large proportion of all cases of environmental pollution crime, and most convictions are due to illegal discharge, rather than actual damage, lacking the actual bodily injury or property damage that anthropocentrism demands. Furthermore, taking animal cruelty as an example, Article 17 of the German Animal Protection Act, Article 28 of the New Zealand Animal Welfare Act, Article 26 of the Swiss Animal Protection Act, and Article 4 of the British Animal Welfare Act all criminalize humans causing serious cruelty to animals. Cruelty to animals is clearly not a violation of human health, but these acts are still included in the criminal circle by criminal law. These phenomena have greatly impacted anthropocentric environmental philosophy [47].

### 3.2. The Theory of Ecocentrism

Against the background of the burgeoning modern environmental protection movement and the great boom in environmental philosophy, the theory of ecocentrism has appeared, trying to push the law system onto a green track [48,49]. The theory holds that although humans have achieved great victories over nature using anthropocentric values, this victory was partial. Since the Industrial Revolution, human activities have often taken the form of harming nature, thereby causing damage to natural values. Now, this damage is threatening human existence. Although there are still different schools of thought in the theory of ecocentrism, in general, the core theory of ecocentrism is that the natural world is an interdependent system, human beings are only one member of this, and all organic individuals are the purpose center of life. Therefore, the natural environment itself is worthy of legal protection [50].

Influenced by this kind of environmental philosophy, many Chinese scholars believe that the key reason for the imperfections in, and ineffective implementation of, China’s environmental crime laws is that they are still influenced by anthropocentrism, and do not have a profound understanding of, and due respect for, the course of nature and the value of natural elements themselves [51]. The current environmental crisis is still severe, and, in this context, this theory has certain advantages. First, the environmental philosophy of ecocentrism has an enlightenment effect on environmental protection. The ecocentrism legal interest theory emphasizes the value and significance of natural ecology, which can shape the public’s ecological awareness to a certain extent, and has an objectively good environmental enlightenment effect [52]. Since the Industrial Revolution, human beings have started on the road of conquest, governance, and even domination of nature. Centuries of excessive pursuit of economic interests and active ignorance of natural laws will ultimately backfire on human beings themselves. Therefore, ecocentrism advocates the high-profile protection of natural ecology, which does have certain practical significance [53]. Second, since natural ecology is regarded as the core value behind the establishment of environmental crime, this theory can solve some of anthropocentrism’s shortcomings. That is to say that although some environmental pollution behaviors do not cause immediate injury to human health, they certainly cause damage to the ecological environment. As long as there is a discharge of pollutants, there must be changes in the characteristics of ecological elements. Therefore, the criminal punishment for polluting the environment can be justified.

However, the shortcomings of ecocentrism are also very obvious. The ecocentrism environmental philosophy has obvious ambiguity and is difficult to apply to a specific legal method. There will be insurmountable logical obstacles in its application to the adjustment of the interactions between nature and people [54]. For instance, some scholars have pointed out that if the natural environment can become the subject of legal relations, then who will determine what rights and obligations should be stipulated, how would nature exercise its own rights and obligations, can the contradiction between man and nature be resolved through negotiation, etc. [55]. Other scholars pointed out that it is difficult for a national criminal legislation to deviate from the development level of productive forces within a specific period. Whether production relations are backward or advanced, once they do not match the productive forces, they will become objects of sublation. This is the unique attribute of environmental crime legislation. The positive significance of an ecocentric environmental philosophy cannot be denied, but it still seems to be a luxury under the current conditions in China [56].

In sum, ecocentric environmental philosophy is an emerging theory in the field of Chinese criminal law; however, due to its more radical theoretical position, the number of advocates of this theory is gradually decreasing.

### 3.3. The Theory of Eco-Anthropocentrism

As the theoretical purposes of anthropocentrism and ecocentrism are at the extremes of conservative and advanced, respectively, and each has insurmountable theoretical flaws, the theory of eco-anthropocentrism attempts to reconcile the two.

In the research on China’s environmental criminal law, eco-anthropocentrism is basically equivalent to weak anthropocentrism. This theory is a common theory in German criminal law academia, and there are many supporters in Japanese criminal law circles. In recent years, more and more Chinese advocates of this theory have appeared.

Eco-anthropocentric environmental philosophy believes that the emergence of a global ecological crisis forces human beings to draw new ethical boundaries for themselves. When dealing with the relationship between people and the natural ecological environment, the pursuit of individual interests must not harm the common interests of human beings. Otherwise, the ecological environment on the earth will no longer be suitable for the survival and development of human beings, and there will no longer be any human interests [57]. In other words, the contemporary ecological crisis generates a modern anthropocentrism that requires the overall and long-term interests of mankind to become the standard when handling the relationship between man and nature. In this context, human beings have put forward a sustainable development strategy to curb environmental degradation and respond to the ecological crisis [58]. Under the guidance of this environmental philosophy, China’s legislation on environmental crimes should fully recognize the connection between human life and the natural environment. The legislation can give the ecological elements (such as air, soil, water, and certain animals and plants) independent legal status when the ecological elements play a role as the basis for human survival [59]. For example, some scholars have pointed out that human interests are the primary concern of environmental criminal law, but criminal law should also be applied to guarantee environmental protection when this is related to human interest.

It seems to be more advantageous to guide the legislation and judiciary regarding environmental crimes in China with an eco-anthropocentric environmental philosophy. The reason for this is that, from the perspective of ecocentrism, any degradation of ecological resources should not be accepted, while the reality is that the legal and rational use of natural resources is not a concern of criminal law. For example, China’s timber demand reached 800 million cubic meters in 2020, and there was still a shortfall of about 200 million cubic meters [60]. The act of legally harvesting these woods will undoubtedly result in the degradation of ecological legal interests but will not be punished by the law. In terms of ecocentrism, the best protection of the natural environment would be that humans do not touch any natural resources. Therefore, the explanatory capability of the ecocentrism theory is very limited, while the eco-anthropocentrism legal interest theory has better explanatory reach. Under the eco-anthropocentrism value, while recognizing the significance of environmental protection, the use of the natural environment within a reasonable range is justified. Therefore, eco-anthropocentrism is not as radical as ecocentrism, and is more in line with current environmental protection needs than anthropocentrism [61].

However, the eco-anthropocentric approach is not perfect and is confronted with very powerful challenges. On the one hand, the distinction between this theory and ecocentrism is not clear, as some people believe that eco-anthropocentrism, in essence, has the same position as ecocentrism. In the case of conflicts between ecological interests and human interests, the same approach as ecocentrism is adopted, namely, giving priority to ecological protection [62]. On the other hand, the legal interest theory of eco-anthropocentrism still has to face the theoretical challenge of vague connotations and the thorny problem of demarcating reasonable boundaries for human use of the environment. Therefore, the theoretical rationality of eco-anthropocentrism does not imply the same reasonableness in practice.

In sum, the three mainstream theories in China at present all have a certain theoretical basis, but all have obvious defects, namely, they cannot accurately reflect the essential characteristics of environmental crime legislation and delineate a fair and reasonable penalty borderline for the judicial practice of environmental crimes. Therefore, it is necessary for us to refer to international experiences to obtain a better reference.

## 4. The International Trend of Environmental Crimes’ Legislation and the Embodiment of Environmental Philosophy

The modern environmental movement originated in the United States and Europe. These environmental protection pioneer countries are more experienced in the application of environmental criminal law [63]. This article will take environmental crime legislation and judicial experience in the United States, Germany, and Japan as examples to illustrate the trends in international environmental criminal law and their environmental philosophy.

### 4.1. United States: One of the Pioneer Countries of Environmental Crime Legislation

Scholars looking at the United States’ environmental crime legislation generally date it back to the “Refuse Act” in 1899 [64]. At the end of the 19th century, the industrial output value of the United States exceeded Britain and became the world’s number one [65]. After entering the 20th century, with the vigorous development of American industry, the environmental crisis in the United States intensified. The Los Angeles optical chemical smoke incident was one of the eight major public environmental incidents in the world in the 20th century [66]. Moreover, in the 1950s, DDT and other highly toxic chemicals were massively used to increase the yield of agricultural products. These toxic chemicals enter the human body through the food chain and induce diseases such as cancer and fetal malformations [67]. The advent of the enlightenment of modern environmental movements, shown in works such as “A Sand County Almanac”, “Silent Spring”, and “The closing circle-Nature, Man and Technology” made the world aware of the importance of environmental protection.

In this context, the United States began to pay attention to the use of criminal law to punish environmental pollution activities after the 1970s. The “Clean Air Act” (1970), the “Clean Water Act” (1972), the “The Resource conservation and Recovery Act” (1976), the “Solid Waste Disposal Act Amendments” (1980), the “the Superfund Amendments and Reauthorization Act” (1986), and other important environmental protection laws with criminal clauses were promulgated or amended, which had a profound impact on the rule of law in worldwide environmental protection history [68]. The criminal clauses in the US Environmental Law are very detailed, and clearly divided into intentional crimes and negligence crimes, felony and minor crimes.

For instance, the “Clean Water Act” sets different levels of penalties based on the subjective malignant and behavioral consequences of the criminal. The mildest is negligent violations. These can be punished by a daily penalty of USD 2500–25,000 or imprisonment below one year; knowing violations can be fined USD 5000–500,000 per day or lead to under three years of imprisonment. Knowing endangerment can be fined no more than USD 250,000 or lead to under 15 years of imprisonment. Secondly, American environmental crime legislation includes behavior that violates environmental management, behavior that enhances environmental risks, and behaviors that cause actual damage. Moreover, people, companies, associations, states, and city governments could become the subjects of environmental crime. This greatly expanded the scope of environmental criminal subjects that can be prosecuted, which greatly strengthened the deterrent effect. Thirdly, to further enforce environmental crimes, the United States also stipulates strict responsibility in certain environmental laws (also known as Superfund Liability) [69,70]. “the Superfund Amendments and Reauthorization Act” in the United States stipulates that, if corporate members discharge dangerous objects or handle dangerous objects in unauthorized places without reporting this, regardless of whether the corporate leaders know, they should be criminally responsible. This strict responsibility legislation reflects the United States’ determination to implement environmental protection and has triggered many academic discussions. In China, discussions on whether to introduce strict responsibilities regarding environmental criminal law have lasted for more than 20 years [71,72,73,74,75].

The U.S. environmental crime legislation has been widely used since the 1980s. At present, the United States has 200–300 environmental crime cases each year [76]. As a pioneer country in the modern environmental movement, the United States is also one of the birthplaces of modern environmental philosophy theory. Admittedly, environmental legislation must consider more reality, so it is impossible to fully accept the theoretical proposition of environmental philosophy. The variety of innovative environmental criminal law systems in the United States and its very strict enforcement more reflect the thought of ecological centrism, which brought huge inspiration to the environmental legislation of other countries [77].

### 4.2. Germany: One of the European Representative Countries with Environmental Crime Legislation

In the environmental movement in the middle and late 20th century, Germany was a pioneer country in Europe. In the 1980s, the German Green Party was founded, which quickly became one of the most influential of the world’s Green Party families, and its influence on German politics has only increased [78]. At present, Germany is considered one of the countries with the strongest environmental awareness. However, this result was not achieved overnight; it took decades of striving towards environmental protection. The legislation of German environmental crimes and its judiciary enforcement has played an irreplaceable role in this process.

After World War II, the industrial development of Germany not only quickly reached the pre-war level, but also made great developments, especially heavy industries such as automobiles, steel, and machinery manufacturing, which became the leaders in the world. However, the rapid development of these industries also brought serious environmental pollution to Germany [79]. In this context, Germany promulgated hundreds of laws and regulations related to environmental protection in a short period of time, but did not achieve a good environmental protection result, instead, the implementation of these environmental laws was in chaos.

German legislators decided to end the chaos. The German legislature passed the 18th Criminal Law Modification Law in March 1980, adding a special sector of environmental crime to the German Criminal Code, which absorbed some of the criminal clauses from the administrative and environmental laws. New crimes, such as water pollution, air pollution, noise pollution, and waste pollution, were created [80]. The addition of environmental crimes to criminal law has completed two major legislative purposes. First, through this amendment, Germany significantly expanded the scope of criminal law to undermine environmental pollution behavior. The second is to reflect the progress in the national environmental protection philosophy through revisions to the law. The trend of moving from anthropocentrism to eco-anthropocentrism has also stimulated national awareness of environmental protection, making the German people more proactive participants in the work of punishing environmental crimes.

The environmental crime legislation that was added in 1980 promoted the unity and standardization of German environmental crime punishment to a certain extent. However, the law still did not resolve the administrative laws’ dependence on environmental crime legislation, which led to certain criticisms. Responding to the concerns of the government and the public, the revised “Anti-Environmental Crime Law” took effect in November 1994, further expanding the scope of environmental crime, aggravating the punishment for environmental crimes, and adding crimes such as land pollution. After the German Criminal Code was revised in 1998, the position regarding environmental crime was transferred to Chapter 29 of the Criminal Code. Since then, the regulations for environmental crimes in Germany have basically stabilized.

In Germany, the environmental criminal law and EU-related environmental crime clauses have been widely applicable. From 1980 to 1990, More than 22,000 criminal cases related to water pollution have been dealt with [81,82]. Since 1980, the environment in Germany has significantly improved [83]. The German academic community has conducted a heated discussion on the environmental philosophy issues in environmental crimes, and human centrism has become the main criterion. However, as a civil-law country, the German academic community believes that, although ecocentrism has a good enlightenment effect, its use as a guiding ideology for environmental crime legislation is risky. Therefore, eco-anthropocentrism has become the mainstream view in Germany for the legislative philosophy regarding environmental crime.

### 4.3. Japan: One of the Asian Representative Countries of Environmental Crime Legislation

Similar to Germany, Japan’s economy also quickly recovered after World War II [84]. Between the 1950s and 1970s, Japan achieved an average annual 10% growth in GDP [85]. However, the rapid development of Japan’s industry has also had a very serious environmental cost, and environmental harm incidents frequently occur [86]. Shockingly, half of the world’s eight major environmental incidents took place in Japan. Following the huge pressure of public opinion, Japan held the 64th Congress in 1970 and formulated 14 laws related to environmental pollution. Among them, the “Law on Punishment on offenses of public hazards involving human health” is Japan’s first formal environmental criminal legislation [87].

Unlike Germany’s practice of incorporating environmental crime legislation into the Criminal Code, Japan’s environmental criminal legislation is a special criminal law, which has many characteristics that differ from the Japanese Criminal Code. First of all, the Japanese Criminal Code only punished persons, but Japan’s environmental criminal law includes enterprises within the scope of punishment. Secondly, Japan’s environmental criminal law also stipulates a different degree of punishment according to different environmental pollution results and the subjective state of the defendant. For example, Article 2 of the law stipulates that, in a factory’s production and operation activities, the intentional discharge of substances that are harmful to human health and cause danger to public life shall be punished with less than 3 years imprisonment or a fine of less than JPY 3 million. If this caused death or serious injury, the sentence could exceed 7 years of imprisonment or fines of less than JPY 5 million. In another example, Article 3 of the law stipulates that if harmful substances are discharged due to negligence in the factory’s daily work, a sentence of less than 2 years imprisonment or a fine of less than JPY 2 million could be sentenced [88]. Thirdly, the Japanese environmental criminal law does not need to prove a specific causal relationship. Instead, it set the provisions of presumption, thereby reducing the court’s difficulty convicting the defendant.

The Japanese environmental criminal law provides a very strong legal guarantee for the improvement of the Japanese environment. The Japanese environmental criminal law is still valued after entering the 21st century. According to statistics, from 2004 to 2008, more than 30,000 cases related to environmental crimes were dealt with [89]. After the strong rectifications in recent decades, Japan has become one of the most beautiful and secure countries in Asia. By controlling the discharge of major pollutants, the content of pollutants such as nitrogen dioxide, sulfur dioxide, carbon dioxide, and granular suspension in the Japanese atmosphere was significantly reduced, and the impact of environmental standards has increased each year. For instance, the water quality of the main rivers in Japan was even higher than that of rivers in European and American countries [90].

In discussions regarding the philosophy of environmental criminal law, Japan, Germany, and China have a similar overall situation: they all have arguments regarding the philosophies of anthropocentrism, ecocentrism, and eco-anthropocentrism. In the Japanese academic community, although supporters of ecocentrism have achieved a strong standing, ecocentrism has not become the mainstream view, and the supporters of eco-anthropocentrism are increasing.

## 5. The Future Development of Environmental Philosophy of China’s Environmental Crime Legislation

After this investigation into relevant international experience, it can be seen that moving from anthropocentrism to ecocentrism is the main trend. The difference between countries is in the degree of ecological centrism. At present, China, as the country with the world’s largest emissions [91], should pay attention to the following aspects when deciding on the future philosophical basis of environmental crime legislation.

First of all, the philosophical choice for China’s environmental crime legislation must learn from international experience, in line with the trend of sustainable development worldwide. As many scholars have criticized, the environmental criminal law established by China in 1997 still regards the natural environment and resources as the object of humans. Environmental criminal laws developed under this concept are very conservative and do not lead to substantial change, so it has not attracted enough attention from judicial organizations and the public. In the late 20th century, major countries in the world adopted the advanced ideas of modern environmental philosophy to guide the formulation of environmental criminal law, and their determination and strength have surpassed China’s environmental criminal law [81]. Regarding crimes of environmental pollution, the United States, Germany, and Japan punished behaviors that merely increase the environmental risks in the 1970s. However, in 1997, China’s environmental criminal law still used damage to human health or property as a must-have factor for conviction. It is gratifying that the establishment of China’s environmental pollution crime, created in 2011, made up for this.

Secondly, China’s environmental crime legislation cannot surpass China’s reality and cannot be completely based on the experience of some environmental pioneer countries. For example, anthropocentrism, as criticized by Chinese and foreign scholars, has many misunderstandings [92]. It is wrong to treat human beings as completely different from other creatures and the center of the universe; this can also be falsified by scientific methods. However, this does not mean that, after tens of thousands of years of evolution, the objective fact that humans possess the advantage of being able to use natural resources can be shaken. It is impossible for humans to be completely separated from their own position, and there is no need to consider their problems and natural laws from the position of “non-anthropocentrism”.

In fact, the emergence of the ecological crisis is an error of anthropocentrism but the result of the lack of environmental justice and environmental fairness. The root cause of the environmental problems caused by human beings is that some people, enterprises, or even some countries have excessively pursued their personal interests and collective interests. This kind of philosophy should be considered within the category of individualism, not anthropocentrism. China’s per capita GDP in 2021 reached USD 12,500, which is still very different from the situation of environmental pioneer countries [93]. Therefore, it is not possible to fully implement an ecocentric philosophy within the legislation and judiciary of environmental crimes in China.

While ecocentrism is too aggressive, anthropocentrism has been criticized almost everywhere. It seems that eco-anthropocentrism is the correct answer. However, the biggest problem of eco-anthropocentrism is that at the macro level, it looks very comprehensive and rational, but eco-anthropocentrism is lacking in the guidance value of legal compilation and policy formulation. The theory does not explain how to deal with the relationship between nature and human beings when the utilization of resources conflict with the interests of animals and plants, which caused the practical value of eco-anthropocentrism to be limited. In other words, eco-anthropocentrism could easily lead legislators and policy makers to be in a state of hesitation; thus, this theory is not suitable for the guidance of the philosophical value of China’s environmental criminal law, and China should surpass the existing, disputed framework. China would absorb the advantages of the current three environmental philosophies and use the philosophy of ecological civilization, which is strongly advocated as a form of value guidance for environmental criminal law. In 2007, the aim of constructing an ecological civilization first appeared in China’s strategic planning [94]. In the last 10 years, the strategic position of ecological civilization has continuously been improved, and it was even written into the Chinese Constitution in 2018. China has publicly stated that the construction of an ecological civilization is a millennium plan for the sustainable development of the Chinese nation [95]. The difference between ecological civilization and anthropocentrism, ecocentrism and other environmental philosophies is that the ecological civilization will be a new form of civilization, guided by the ecological sciences and surpassing the industrialization civilization. Anthropocentrism, ecocentrism, and other environmental philosophies are discussions in the field of philosophy; the ecological civilization proposed by China includes four dimensions: economy, politics, society, and culture. The ecological civilization will encompass environmental justice, with the goal of developing ecological communities, and reasonably coordinating the relationship between man and nature [96].

Ecological civilization is not just an environmental philosophy or environmental ethics, but a social development theory that integrates multi-dimensional thinking and has the value of better guiding practice. Reference [97] Compared with Western environmental philosophy, ecological civilization theory has the following main characteristics.

Primarily, the core proposition of ecological civilization theory is that a good ecological environment is a kind of productive force itself [98]. Therefore, protecting the environment and economic development is not an opposition, and the environmental protection policy of a country should be closely integrated with economic policies. In fact, whether it is anthropocentrism, ecocentrism, or eco-anthropocentrism, the essence of their thoughts stems from the dichotomy of subject–object [99]. However, the theory of ecological civilization believes that a good ecological environment requires science and technology upgrades, industrial transformation, and capital investment. This is an important driving force for the economic development of a country. Human beings can achieve a win–win situation for environmental protection and economic development [100].

Moreover, the theory of ecological civilization pointed out that a good ecological environment is the fairest public product and the most inclusive people’s livelihood, which reflects the absorption and development of environmental justice and environmental politics [101]. With the continuous improvement of China’s social development and people’s living standards, the status of the ecological safety and happiness has become prominent, which is related to the stability of social order and governance. In order to better protect the environment, we need to introduce the corresponding construction goals and assessment systems for the governors [102]. Therefore, the theory of ecological civilization obviously has better practical value and can provide a clear direction for the government’s environmental protection work.

Finally, the theory of ecological civilization proposes that the strictest system and the rigorous rule of law are reliable guarantees for the construction of ecological civilization [103]. The “strictest system” means that the existing system must be truly implemented. In the construction of ecological civilization, whether it is ordinary people or government leaders, as long as they have the behavior of polluting or destroying natural environment, they must be punished by law [104,105]. Therefore, it is urgent to update and improve the corresponding institutional system in accordance with the needs of ecological civilization construction, so that the construction of ecological civilization can truly “rely on the law.”

In summary, there is a big difference between ecological civilization theory and environmental philosophy; the environmental philosophy guiding China’s establishment of environmental crimes should absorb more reasonable thoughts on the theory of ecological civilization. This is not only more in line with China’s current conditions, but also can bring new perspectives to the overseas research on environmental studies.

## 6. Conclusions

The environmental crisis has spawned modern environmental philosophies, making countries around the world recognize the extreme importance of environmental protection. As laws such as civil law and administrative environmental law can only fine the subject that pollutes the environment, many individuals or enterprises take the fines and do not pay attention to optimizing their environmental protection. Since the 1960s and 1970s, the emergence of environmental criminal law has greatly improved this phenomenon, strengthened the deterrents in environmental protection laws, and significantly improved people’s environmental awareness. At present, the inherent value of the natural environment is increasingly being recognized, and China is no exception.

Since the 1990s, the relevant experience with environmental criminal legislation in Germany and Japan has become the main object of China’s environmental criminal legislation. Therefore, the discussion of China’s environmental philosophy regarding environmental crime legislation is also very similar to the situation in these two countries and has long hovered between anthropocentrism, ecocentrism, and eco-anthropocentrism. Complete anthropocentrism and complete ecocentrism seem to be a bit radical, but eco-anthropocentrism is the perfect answer. Therefore, China should consider going beyond the arguments surrounding these three philosophies and start to attach importance to the theory of ecological civilization that China has vigorously advocated in recent years as the value basis for environmental crime legislation. This would not only be more in line with the requirements of the Chinese Constitution and the rule of law but it would also provide new ideas for the study of environmental crime and promote the continuous development of human environmental protection.

## Data Availability

Not applicable.
